# Supplementary material for: Myasthenia gravis-specific aberrant neuromuscular gene expression by medullary thymic epithelial cells in thymoma
Source: Nat Commun. 2022 Jul 22;13:4230. doi: 10.1038/s41467-022-31951-8 (PMC9305039; doi:10.1038/s41467-022-31951-8)
Supplement: Supplementary file 1 — Supplementary Information [file 41467_2022_31951_MOESM1_ESM.pdf]

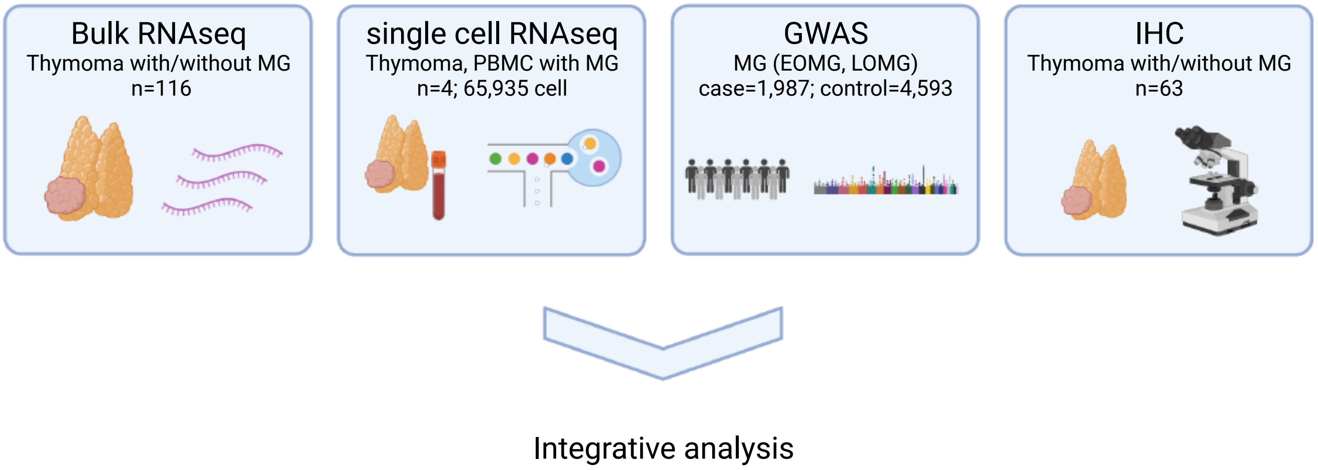

Supplementary Figure 1 Schematic view of the analysis flow.

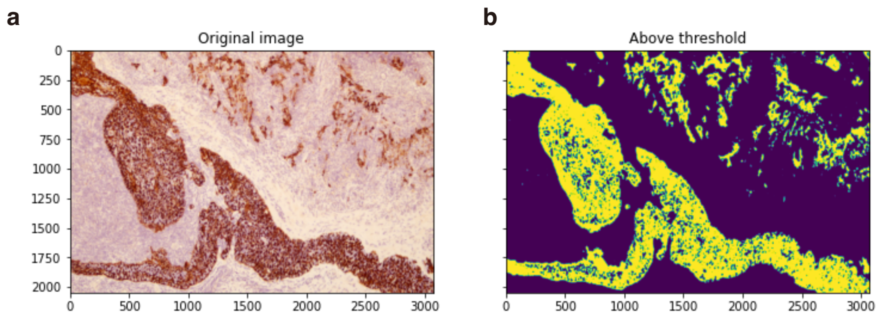

Supplementary Figure 2 DAB signal segmentation and quantification. (a) An original DAB image under x10 objective. (b) The DAB positive area was determined automatically. We split color into three channels; DAB, hematoxyline, and eosin, then discriminated DAB positive area with an empirical threshold.

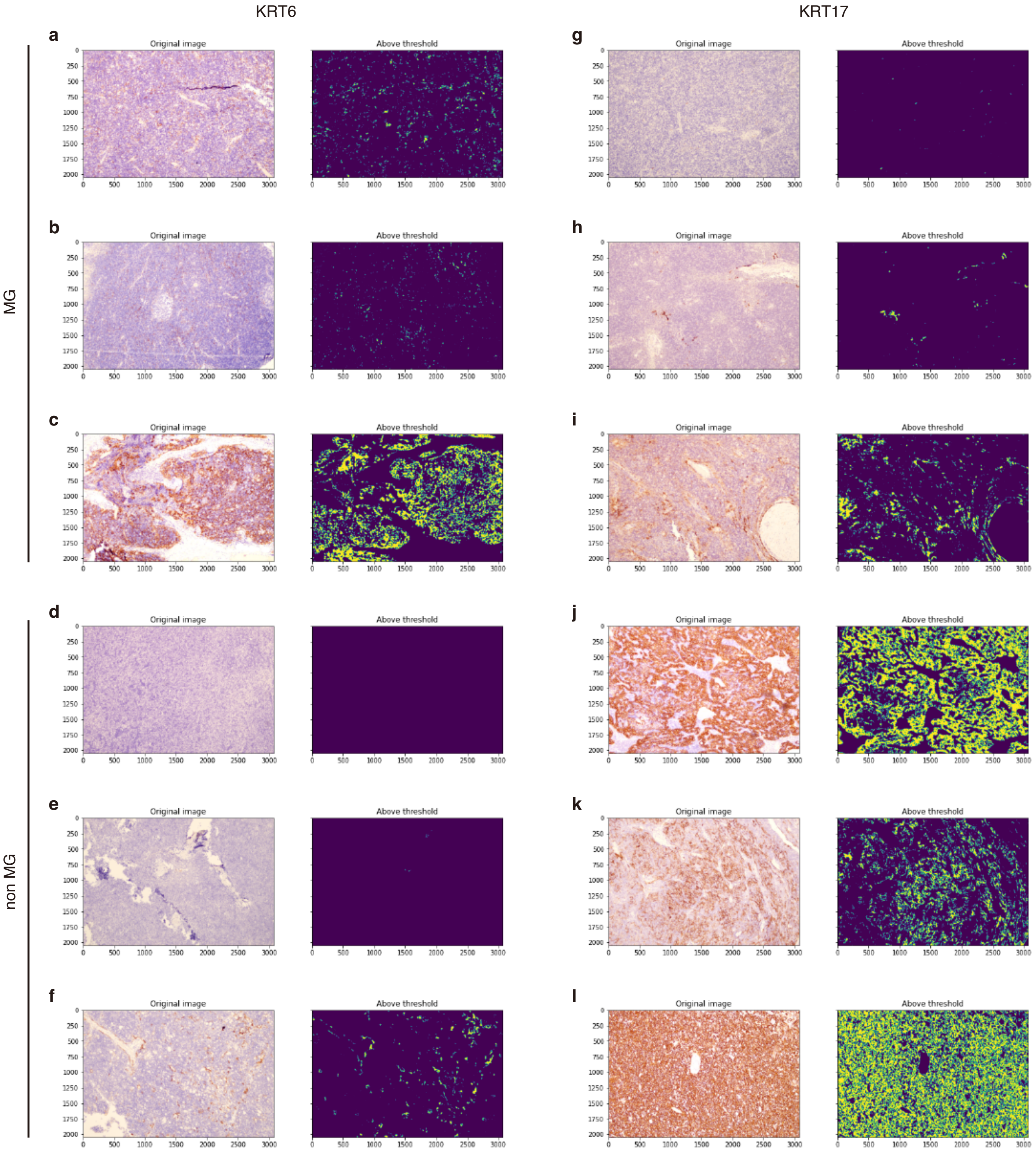

Supplementary Figure 3 The representative images of KRT6 (**a**,**b**,**c**,**d**,**e**,**f**) and KRT17 (**g**,**h**,**i**,**j**,**k**,**l**) staining in MG (**a**,**b**,**c**,**g**,**h**,**i**) and non-MG (**d**,**e**,**f**,**j**,**k**,**l**) thymoma under x10 objective. **a** and **g**; **b** and **h**; **c** and **i**; **d** and **j**; **e** and **k**; **f** and **l** are tissue sections from corresponding donors.

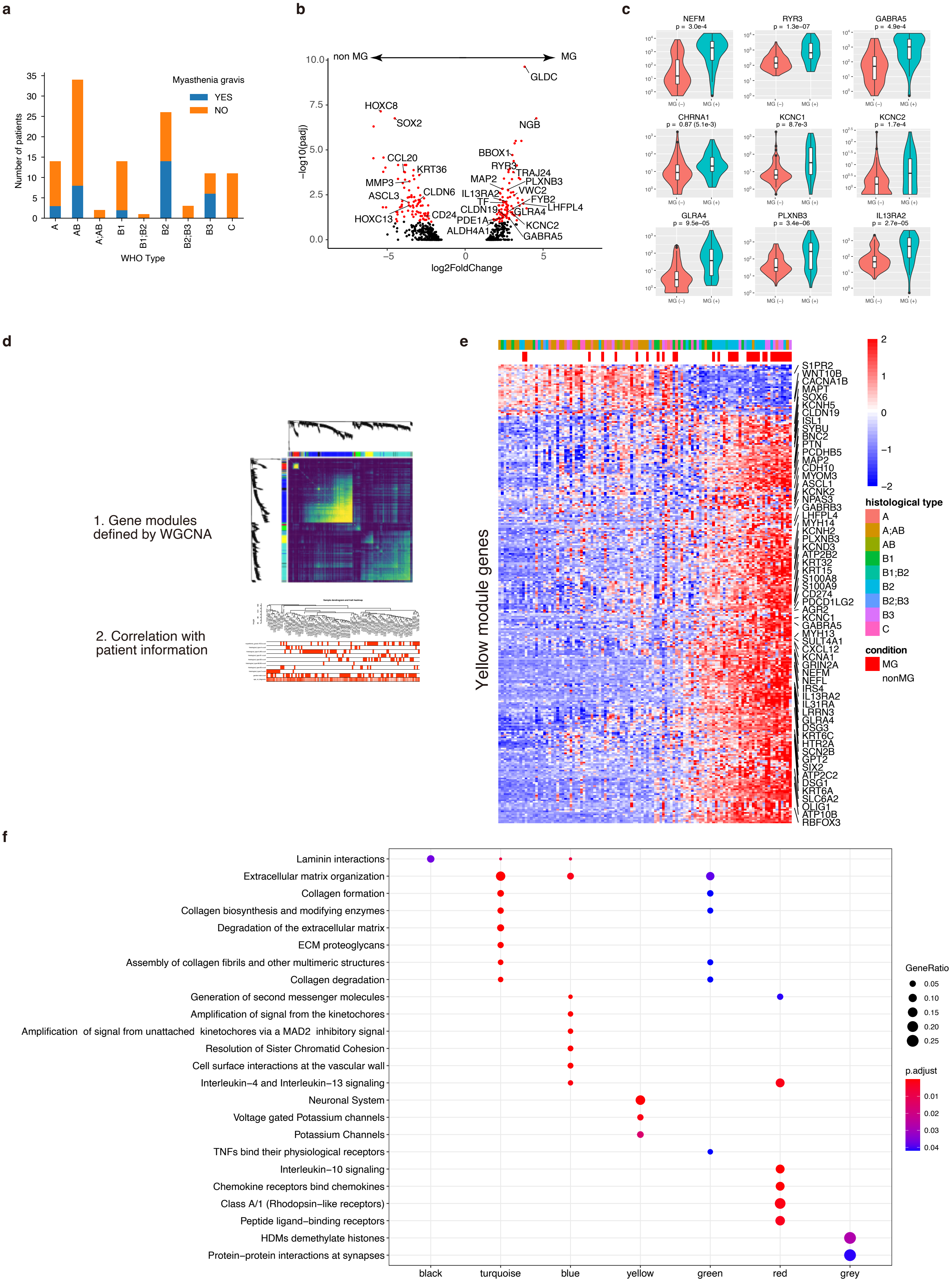
a
Myasthenia gravis
YES
NO
Number of patients
35
30
25
20
15
10
5
0
A
AB
A;AB
B1
B1;B2
B2
B2;B3
B3
C
WHO Type
b
non MG
MG
−log10(padj)
10.0
7.5
5.0
2.5
0.0
−5
0
5
log2FoldChange
GLDC
HOXC8
SOX2
NGB
BBOX1
CCL20
RYR3
KRT36
TRAJ24
MAP2
PLXNB3
MMP3
VWC2
IL13RA2
ASCL3
CLDN6
FYB2
TF
LHFPL4
CLDN19
CD24
GLRA4
PDE1A
HOXC13
KCNC2
ALDH4A1
GABRA5
c
NEFM
p = 3.0e-4
RYR3
p = 1.3e−07
GABRA5
p = 4.9e-4
CHRNA1
p = 0.87 (5.1e-3)
KCNC1
p = 8.7e-3
KCNC2
p = 1.7e-4
GLRA4
p = 9.5e−05
PLXNB3
p = 3.4e−06
IL13RA2
p = 2.7e−05
MG (−)
MG (+)
d
1. Gene modules
defined by WGCNA
2. Correlation with
patient information
e
Yellow module genes
S1PR2
WNT10B
CACNA1B
MAPT
SOX6
KCNH5
CLDN19
ISL1
SYBU
BNC2
PTN
PCDHB5
MAP2
CDH10
MYOM3
ASCL1
KCNK2
NPAS3
GABRB3
LHFPL4
MYH14
KCNH2
PLXNB3
KCND3
ATP2B2
KRT32
KRT15
S100A8
S100A9
CD274
PDCD1LG2
AGR2
KCNC1
GABRA5
MYH13
SULT4A1
CXCL12
KCNA1
GRIN2A
NEFM
NEFL
IRS4
IL13RA2
IL31RA
LRRN3
GLRA4
DSG3
KRT6C
HTR2A
SCN2B
GPT2
SIX2
ATP2C2
DSG1
KRT6A
SLC6A2
OLIG1
ATP10B
RBFOX3
2
1
0
−1
−2
histological type
A
A;AB
AB
B1
B1;B2
B2
B2;B3
B3
C
condition
MG
nonMG
f
Laminin interactions
Extracellular matrix organization
Collagen formation
Collagen biosynthesis and modifying enzymes
Degradation of the extracellular matrix
ECM proteoglycans
Assembly of collagen fibrils and other multimeric structures
Collagen degradation
Generation of second messenger molecules
Amplification of signal from the kinetochores
Amplification of signal from unattached kinetochores via a MAD2 inhibitory signal
Resolution of Sister Chromatid Cohesion
Cell surface interactions at the vascular wall
Interleukin−4 and Interleukin−13 signaling
Neuronal System
Voltage gated Potassium channels
Potassium Channels
TNFs bind their physiological receptors
Interleukin−10 signaling
Chemokine receptors bind chemokines
Class A/1 (Rhodopsin−like receptors)
Peptide ligand−binding receptors
HDMs demethylate histones
Protein−protein interactions at synapses
black
turquoise
blue
yellow
green
red
grey
GeneRatio
0.05
0.10
0.15
0.20
0.25
p.adjust
0.01
0.02
0.03
0.04

Supplementary Figure 4 Global profiling of TCGA thymoma bulk RNA-seq dataset.

a, Bar plot of patient distribution partitioned by WHO classification and MG status. The MG complication rates were 21.4% in type A, 23.5% in type AB, 0% in type A;AB, 14.3% in type B1, 0% in type B1;B2, 53.8% in type B2, 0% in type B2;B3, 54.5% in type B3, and 0% in type C. b, A volcano plot showing adjusted P-value and log2 fold change for differential testing of genes between MG and non-MG patients in bulk RNA-seq of thymoma. Red dots represent statistically significant genes (Padj < 0.1, |log2 fold change| > 1). Multiple testing correction was performed by DESeq2. c, Violin plots of DESeq2 normalized expression for MG-specific genes. Adjusted P-value by DESeq2 and for CHRNA1 P-value calculated by a two-sided Mann-Whitney U test (in parentheses) are shown. Box plots show IQRs and whiskers show the maximum or minimum value in the dataset excluding outliers (Q3+1.5×IQR or Q1-1.5×IQR). (n=116) d, Workflow for Weighted Correlation Network Analysis (WGCNA). In WGCNA analysis, we first defined gene modules based on gene-wise expression correlation, then integrated clinical information. e, Heatmap showing standardized expression of genes in the yellow module. Samples were sorted by eigengene value. WHO classification and MG status are shown at the top of the heatmap. f, REACTOME pathways enriched in each module.

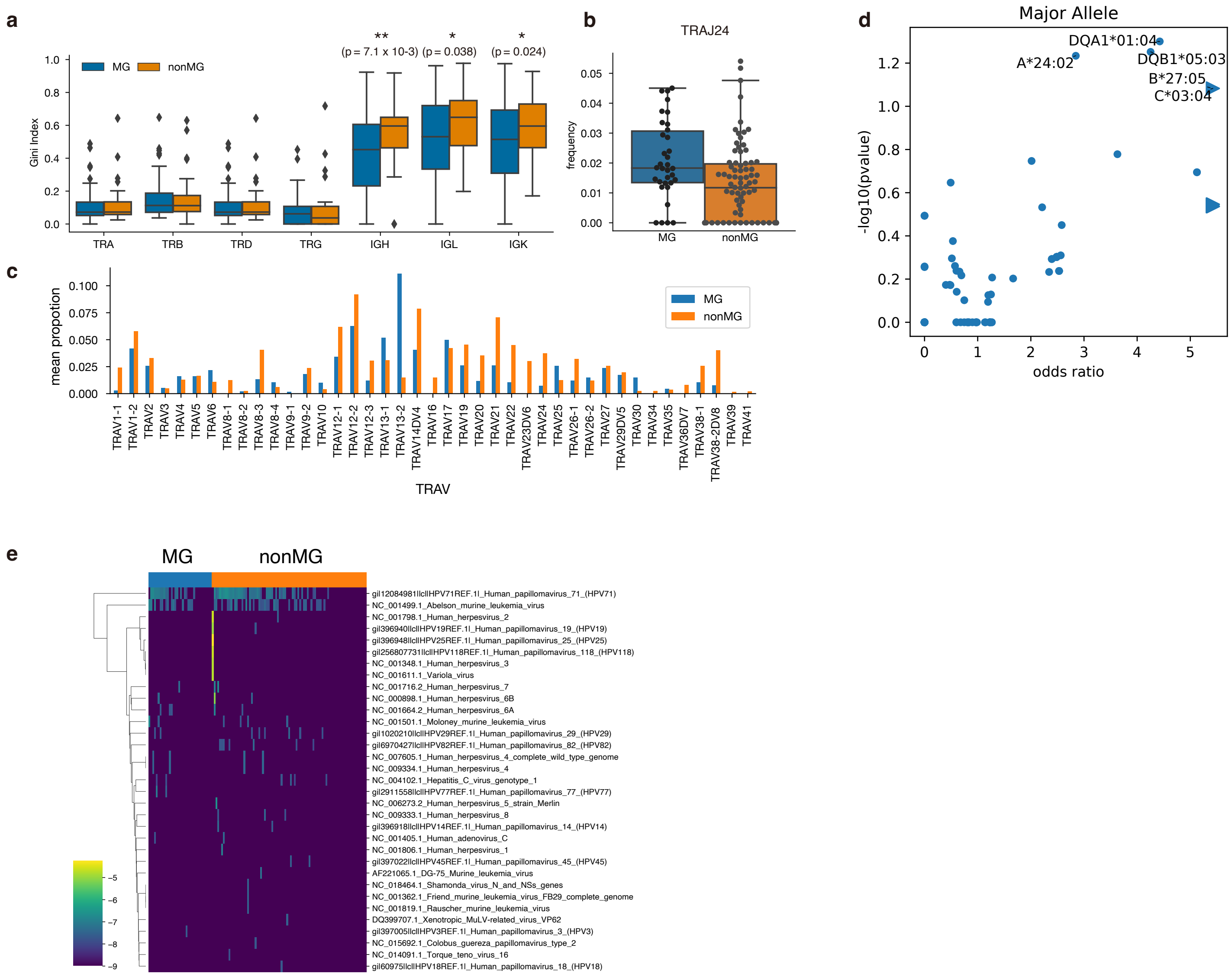

Supplementary Figure 5 TCGA thymoma bulk RNA-seq dataset (n=116) elucidated Immune characteristics of MG.

a, Amino acid sequences diversity of immunoreceptors in CDR3 regions. The Gini index was used as an index of the complexity of repertoires in each group, and differences between MG and non-MG were tested using a two-sided Mann-Whitney U test. *P < 0.05, **P < 0.01. Box plots show IQRs and whiskers show the maximum or minimum value in the dataset excluding outliers (Q3+1.5×IQR or Q1-1.5×IQR). b, Box plot showing the frequency of TRAJ24 in MG- and non-MG- thymoma. Box plots show IQRs and whiskers show the maximum or minimum value in the dataset excluding outliers (Q3+1.5×IQR or Q1-1.5×IQR). Adjusted p-value was calcurated by The BH procedure. c, Bar plot of the frequency of TRAJ genes paired with TRAJ24. TRAV13-2 was 7.50 times more frequent but not statistically significant. d, Volcano plot showing association of HLA major alleles with MG. Data were analyzed using a two-sided Fisher' s exact test. e, Heatmap of detected viruses in thymoma. The color indicates the number of transcripts mapped for each virus.

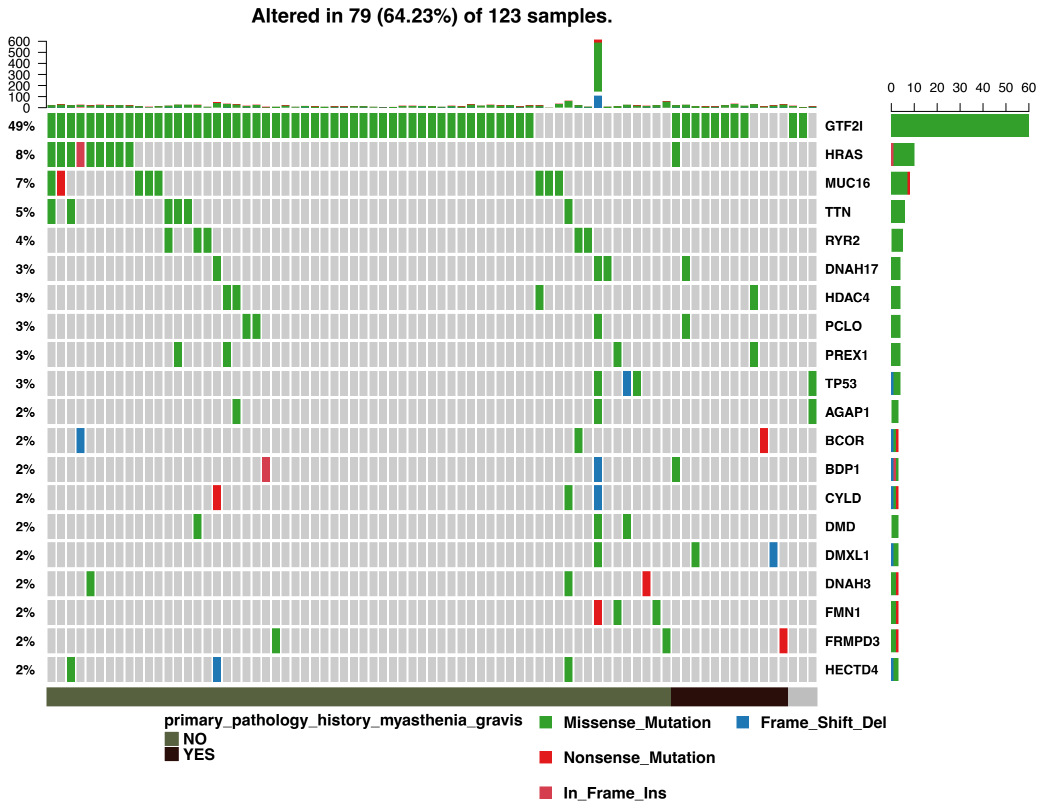

Supplementary Figure 6 No somatic mutation in TCGA thymoma was associated with myasthenia gravis.

Somatic mutations detected within TCGA thymoma samples with and without MG. Rows show observed variants aggregated by genes, and columns show individuals with MG status (below). The color represents the type of mutation. The frequency of the gene was mutated (right), and the abundance of mutation in each individual (top) is also shown.

Epthelial cells

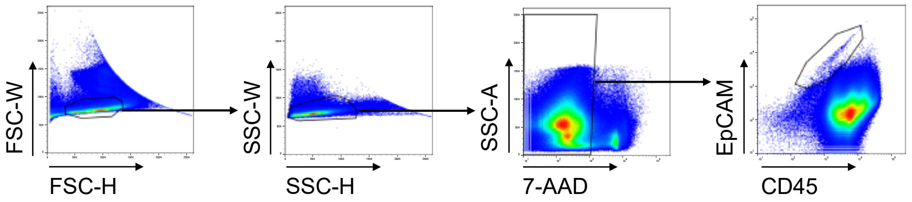

Immune cells (thymus)

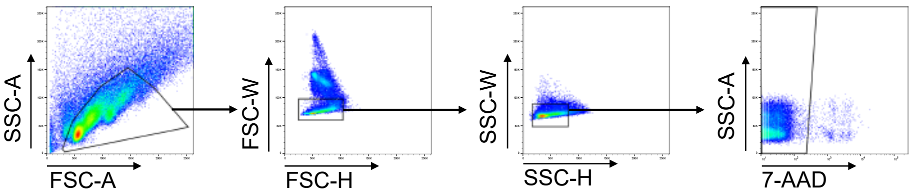

Supplementary Figure 7 FACS gating for single-cell experiments.

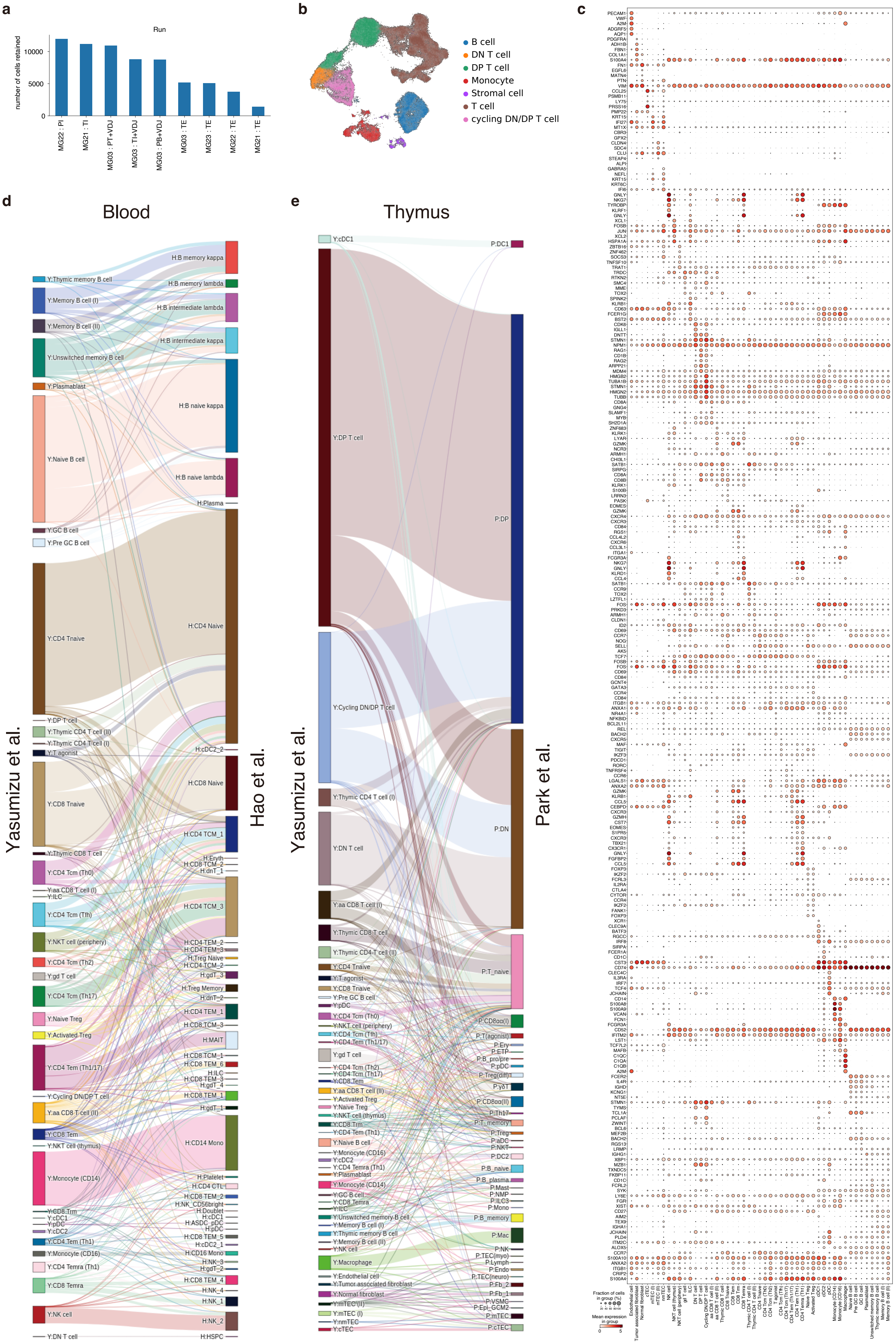
a
Run
number of cells retained
10000
5000
0
MG22 : PI
MG21 : TI
MG03 : PT+VDJ
MG03 : TI+VDJ
MG03 : PB+VDJ
MG03 : TE
MG23 : TE
MG22 : TE
MG21 : TE
b
B cell
DN T cell
DP T cell
Monocyte
Stromal cell
T cell
cycling DN/DP T cell
c
Fraction of cells in group (%)
Mean expression in group
d
Blood
Yasumizu et al.
Hao et al.
e
Thymus
Yasumizu et al.
Park et al.

Supplementary Figure 8 Confirmation of scRNAseq annotation and embedding.

a, The number of cells recovered from each sample. We collected cells from four individuals with MG and thymoma. The abbreviation after the underscore in the sample ID indicates the source of the sample. TI: Thymoma immune cells, TE: Thymoma Epithelial cells, PI: Periphery immune cells, PT: Periphery CD4+ T cells, PB: Periphery CD19+ B cells. VDJ indicates 10x Genomics 5' +VDJ kit; otherwise, 10x Genomics 3' GEM v3. b, The major categories on UMAP embedding. c, Detailed dot plot depicting signature genes' mean expression levels and percentage of cells expressing them across clusters. d,e, Sankey diagrams showing cells aligned to each other in the thymus (d) and blood (e) of healthy individuals and our data set.

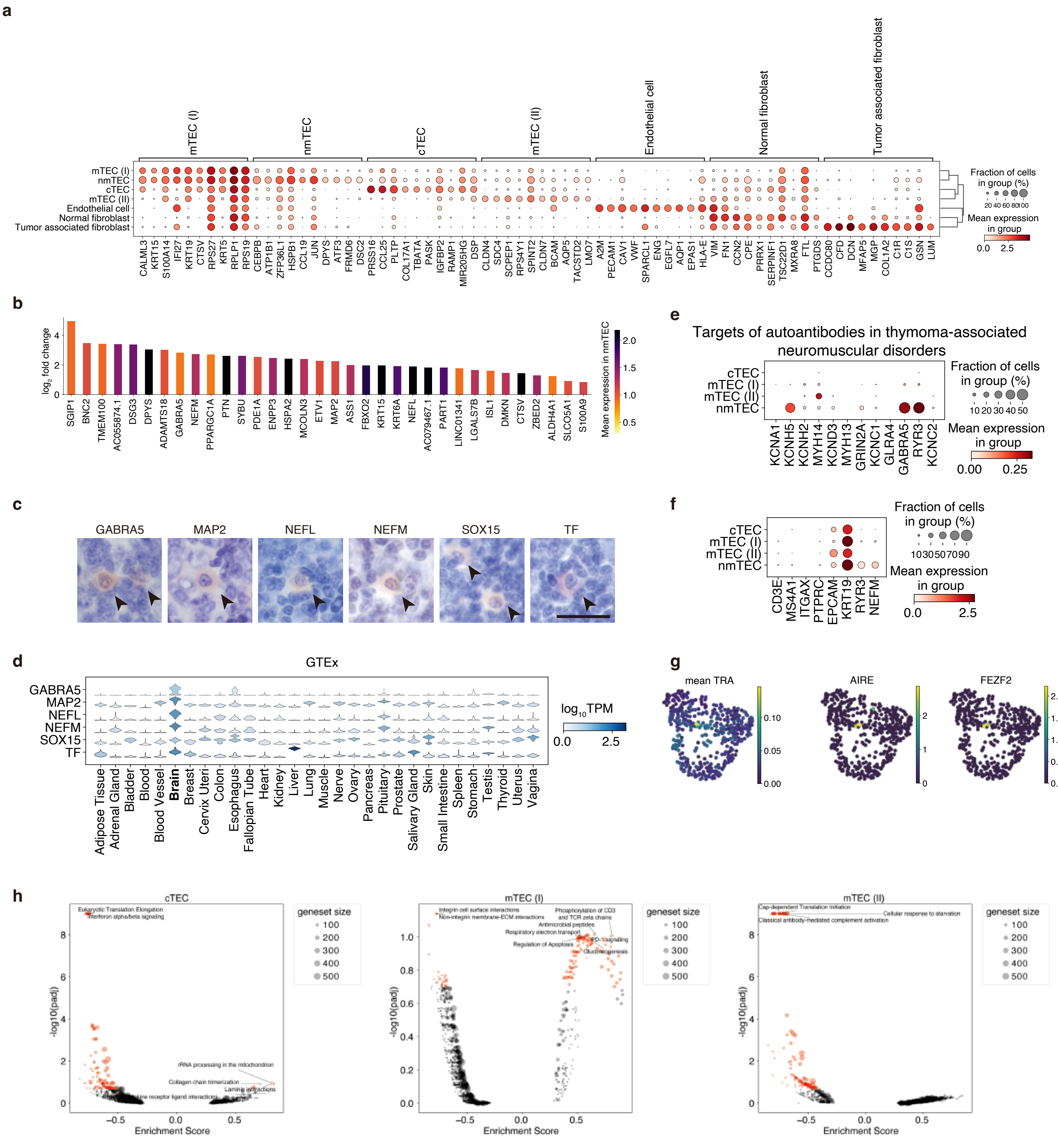

Supplementary Figure 9 Detailed single-cell profiling of TEC cells.

a, Dot plot of signature gene expression in stromal cell clusters. b, Bar plot for the yellow module genes with log2 fold change (MG vs. nonMG in TCGA bulk RNAseq dataset) > 1 and mean expression nmTEC > 0.3 in nmTECs. log2(fold change) was calculated by comparison with other TEC clusters. Mean expression in nmTEC was represented by color. c, Immunohistochemistry of the yellow module genes. Scale bar: 20μm. d, Violine plots of the yellow module genes' expressions across tissues in GTEx samples. e,f, Dot plot of gene expression of targets of autoantibodies in thymoma-associated neuromuscular disorders (e) and marker genes (f) in TEC clusters. g, UMAP embedding of mean expression of tissue-restricted antigens (TRAs) defined using GTEx bulk RNA-seq from systemic organs (left), and essential genes for TRA regulation; AIRE (middle), FEZF2 (right). h, Volcano plot showing REACTOME gene sets enriched in TEC clusters (Supplementary Table 12-14). Multiple testing corrections were performed by the BH procedure.

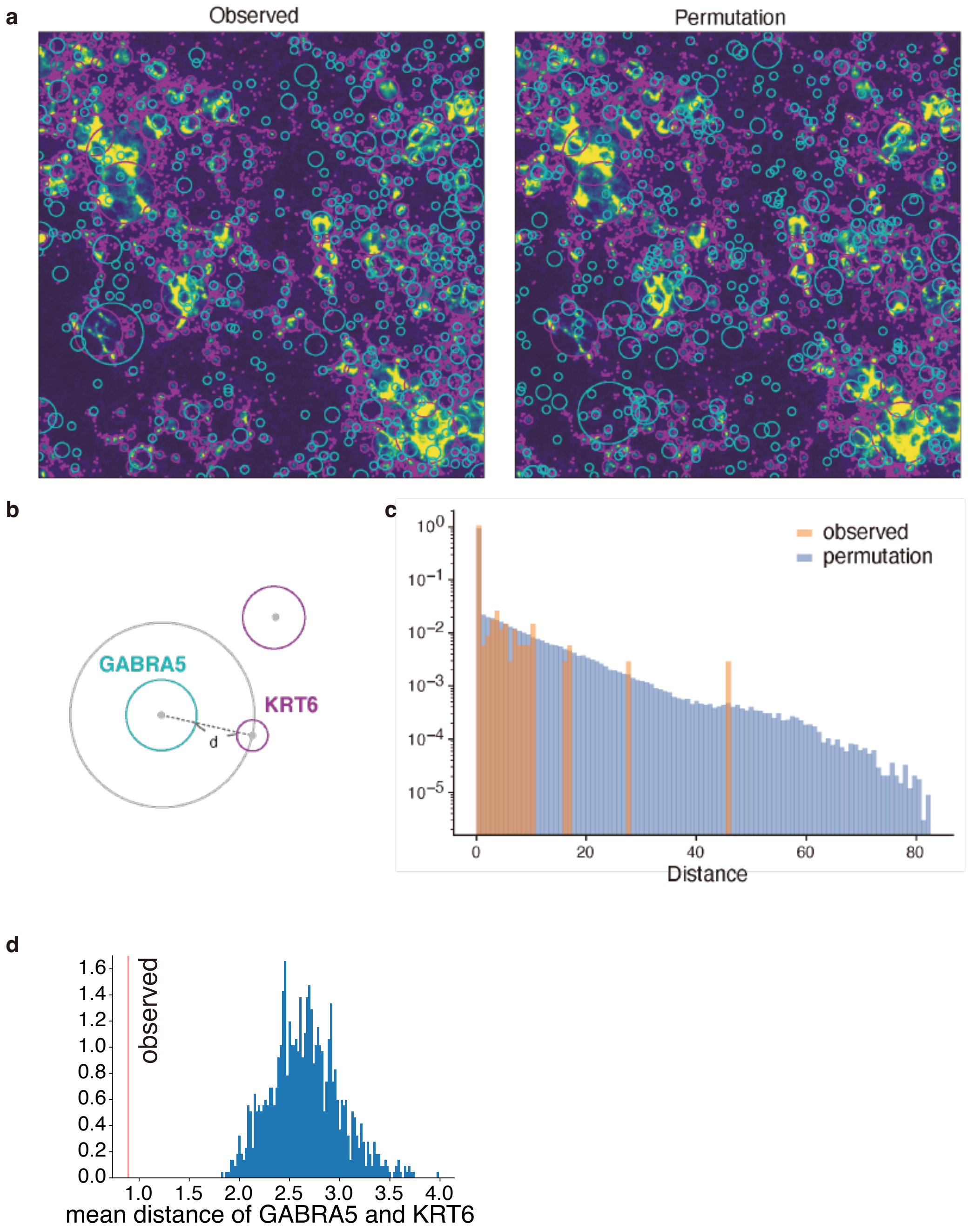

Supplementary Figure 10 GABRA5 and KRT6 were co-localized. (a) Signal blobs and permutated blobs for GABRA5 (blue) and KRT6 (purple). We conducted blob detection using immunofluorescence images stained GABRA5 and KRT6 (left). We also permutated blobs whose sizes and numbers were the same as the original image for 1000 sets (right). (b) The definition of distances of blobs. We measured the distance from GABRA5 blobs to the nearest KRT6 blobs. (c) The histogram is representing distances for observed blobs (orange) and permutated blobs (blue). (d) Mean distance of GABRA5 and KRT6 (a red line) and these for 1000 sets of permutation. The observed mean distance was smaller than all of the permutation sets.

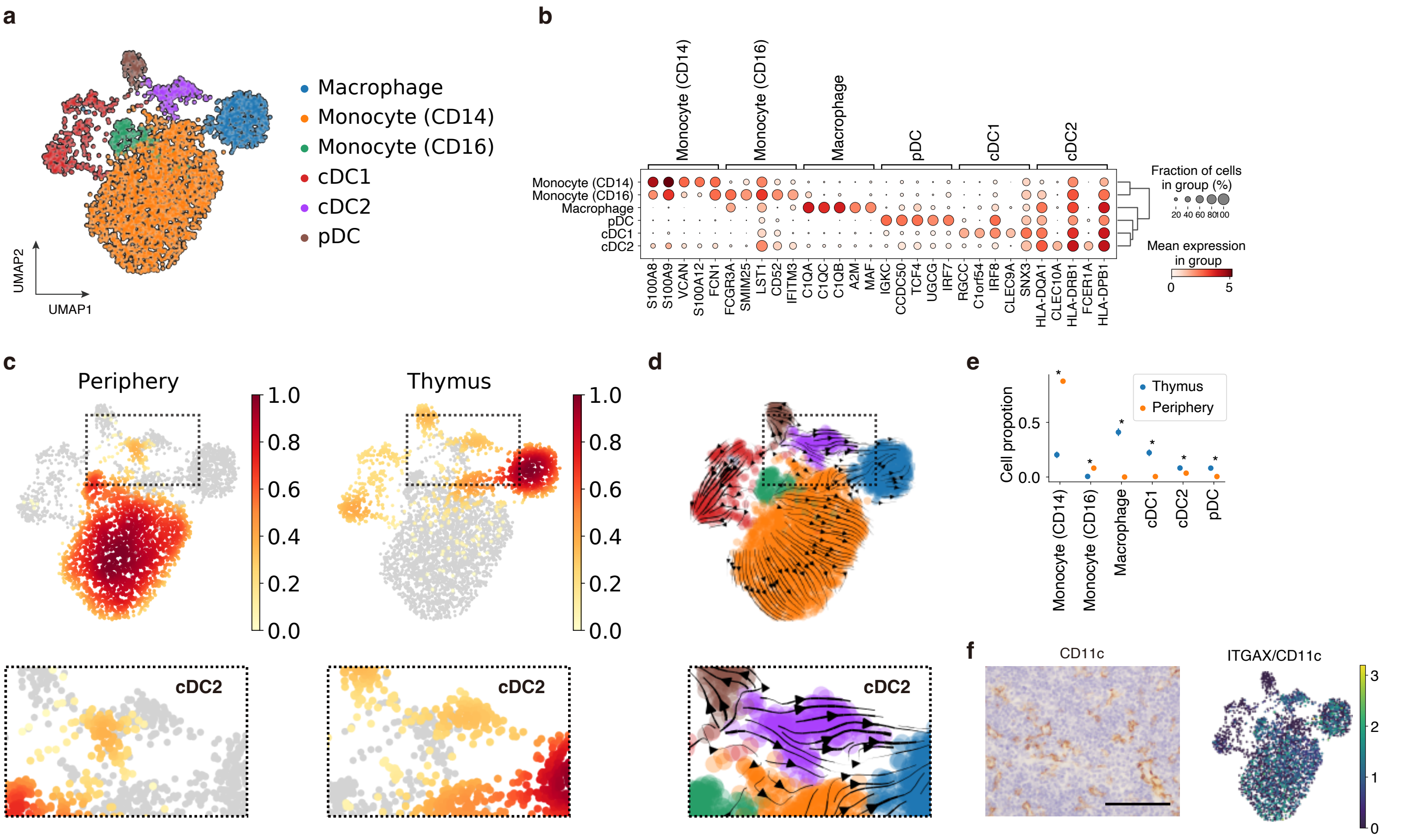

Supplementary Figure 11 single-cell profiling of myeloid cells.

a, UMAP embedding for myeloid cell clusters of thymoma and peripheral blood. b, Dot plot of gene expression of marker genes of each myeloid cell cluster. c,d, Density plots showing myeloid cell accumulation in the periphery (left) and thymus (right) (c) and RNA velocity in myeloid cells (d). Upper figures show the global picture, and lower images show the local picture focusing on cDC2s. e, Cell proportion of each myeloid cell cluster in thymoma and peripheral blood. Error bars show 98% highest density interval. Center points represent the mean of the posterior distributions. Periphery n=2, Thymoma n=4. *FDR < 0.05. f, Representative DAB staining for CD11c in MG-type thymoma stained (left) and UMAP embedding of ITGAX (CD11c) expression. Scale bar: 100μm.

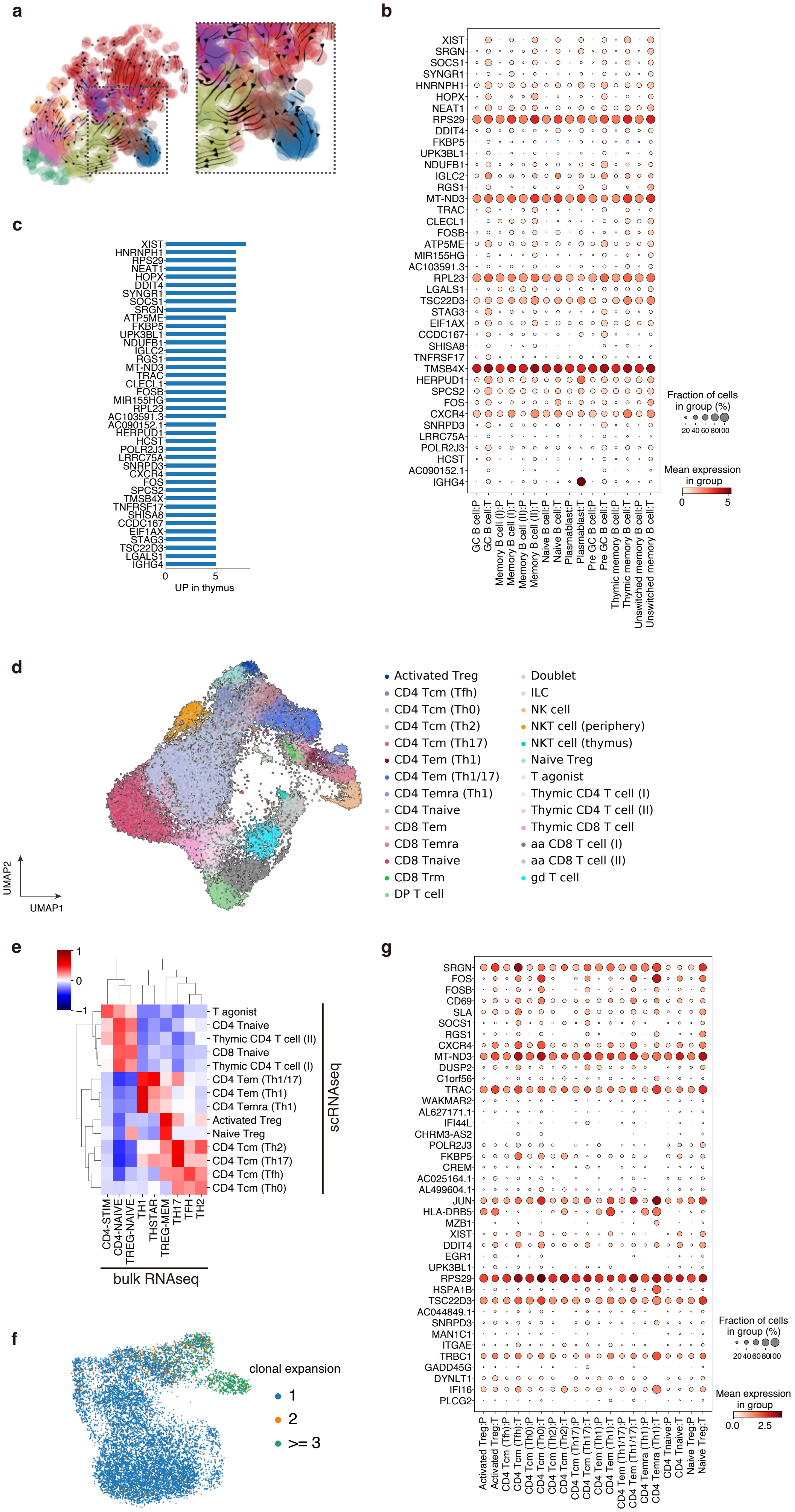
a
b
XIST
SRGN
SOCS1
SYNGR1
HNRNPH1
HOPX
NEAT1
RPS29
DDIT4
FKBP5
UPK3BL1
NDUFB1
IGLC2
RGS1
MT-ND3
TRAC
CLECL1
FOSB
ATP5ME
MIR155HG
AC103591.3
RPL23
LGALS1
TSC22D3
STAG3
EIF1AX
CCDC167
SHISA8
TNFRSF17
TMSB4X
HERPUD1
SPCS2
FOS
CXCR4
SNRPD3
LRRC75A
POLR2J3
HCST
AC090152.1
IGHG4
GC B cell:P
GC B cell:T
Memory B cell (I):P
Memory B cell (I):T
Memory B cell (II):P
Memory B cell (II):T
Naive B cell:P
Naive B cell:T
Plasmablast:P
Plasmablast:T
Pre GC B cell:P
Pre GC B cell:T
Thymic memory B cell:P
Thymic memory B cell:T
Unswitched memory B cell:P
Unswitched memory B cell:T
Fraction of cells in group (%)
20 40 60 80 100
Mean expression in group
0 5
c
XIST
HNRNPH1
RPS29
NEAT1
HOPX
DDIT4
SYNGR1
SOCS1
SRGN
ATP5ME
FKBP5
UPK3BL1
NDUFB1
IGLC2
RGS1
MT-ND3
TRAC
CLECL1
FOSB
MIR155HG
RPL23
AC103591.3
AC090152.1
HERPUD1
HCST
POLR2J3
LRRC75A
SNRPD3
CXCR4
FOS
SPCS2
TMSB4X
TNFRSF17
SHISA8
CCDC167
EIF1AX
STAG3
TSC22D3
LGALS1
IGHG4
0 5
UP in thymus
d
UMAP2
UMAP1
Activated Treg
CD4 Tcm (Tfh)
CD4 Tcm (Th0)
CD4 Tcm (Th2)
CD4 Tcm (Th17)
CD4 Tem (Th1)
CD4 Tem (Th1/17)
CD4 Temra (Th1)
CD4 Tnaive
CD8 Tem
CD8 Temra
CD8 Tnaive
CD8 Trm
DP T cell
Doublet
ILC
NK cell
NKT cell (periphery)
NKT cell (thymus)
Naive Treg
T agonist
Thymic CD4 T cell (I)
Thymic CD4 T cell (II)
Thymic CD8 T cell
aa CD8 T cell (I)
aa CD8 T cell (II)
gd T cell
e
1
0
−1
T agonist
CD4 Tnaive
Thymic CD4 T cell (II)
CD8 Tnaive
Thymic CD4 T cell (I)
CD4 Tem (Th1/17)
CD4 Tem (Th1)
CD4 Temra (Th1)
Activated Treg
Naive Treg
CD4 Tcm (Th2)
CD4 Tcm (Th17)
CD4 Tcm (Tfh)
CD4 Tcm (Th0)
scRNAseq
CD4-STIM
CD4-NAIVE
TREG-NAIVE
TH1
THSTAR
TREG-MEM
TH17
TFH
TH2
bulk RNAseq
f
clonal expansion
1
2
>= 3
g
SRGN
FOS
FOSB
CD69
SLA
SOCS1
RGS1
CXCR4
MT-ND3
DUSP2
C1orf56
TRAC
WAKMAR2
AL627171.1
IFI44L
CHRM3-AS2
POLR2J3
FKBP5
CREM
AC025164.1
AL499604.1
JUN
HLA-DRB5
MZB1
XIST
DDIT4
EGR1
UPK3BL1
RPS29
HSPA1B
TSC22D3
AC044849.1
SNRPD3
MAN1C1
ITGAE
TRBC1
GADD45G
DYNLT1
IFI16
PLCG2
Activated Treg:P
Activated Treg:T
CD4 Tcm (Tfh):P
CD4 Tcm (Tfh):T
CD4 Tcm (Th0):P
CD4 Tcm (Th0):T
CD4 Tcm (Th2):P
CD4 Tcm (Th2):T
CD4 Tcm (Th17):P
CD4 Tcm (Th17):T
CD4 Tem (Th1):P
CD4 Tem (Th1):T
CD4 Tem (Th1/17):P
CD4 Tem (Th1/17):T
CD4 Temra (Th1):P
CD4 Temra (Th1):T
CD4 Tnaive:P
CD4 Tnaive:T
Naive Treg:P
Naive Treg:T
Fraction of cells in group (%)
20 40 60 80 100
Mean expression in group
0.0 2.5

Supplementary Figure 12 Detailed single-cell profiling of B cells and T cells.

a, RNA velocity of intrathymic B cell in the global picture and local picture focusing on GC B cells and the neighboring cells. b, Dot plot of B cell gene expression of genes preferentially expressed in thymus across cell type. In column labels, P: peripheral blood, T: thymoma. c, Bar plot of thymus specific genes across B cell clusters ranked by the number of cell types where each gene was upregulated ($P_{adj} < 0.05$ and log2 fold change > 1) in B cells. d, UMAP embedding for T cell clusters except for DN and cycling DN/DP T cells of thymoma and peripheral blood. e, Heatmap of the correlation between CD4+ T cell clusters defined by our scRNAseq dataset and bulk RNA-seq sorted from peripheral blood established by the DICE (Database of Immune Cell Expression, Expression quantitative trait loci (eQTLs) and Epigenomics) project. f, UMAP embedding depicting the size of clonotypes. g, Dot plot of CD4+ T cell gene expression of genes preferentially expressed in thymus across cell type. In column labels, P: peripheral blood, T: thymoma.

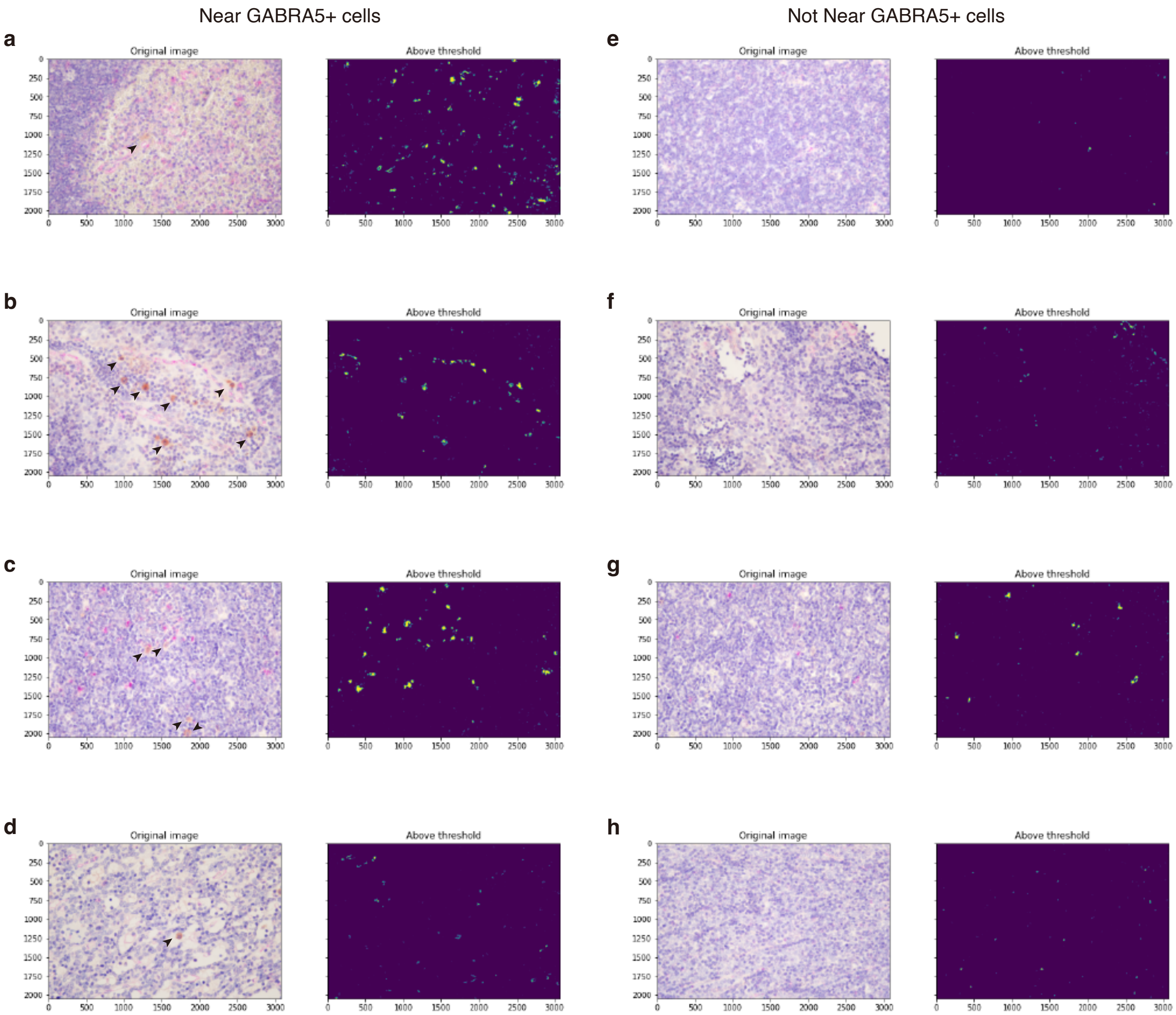

Supplementary Figure 13 The representative images of CD31+ (purple) endothelial cells (left) and detected CD31 signals (right) near (**a**-**d**) and not near (**e**-**h**) GABRA5+ (DAB) cells under x40 objective. **a** and **e**; **b** and **f**; **c** and **g**; **d** and **h** are tissue sections from corresponding donors. Arrowheads represent GABRA5+ cells.

a

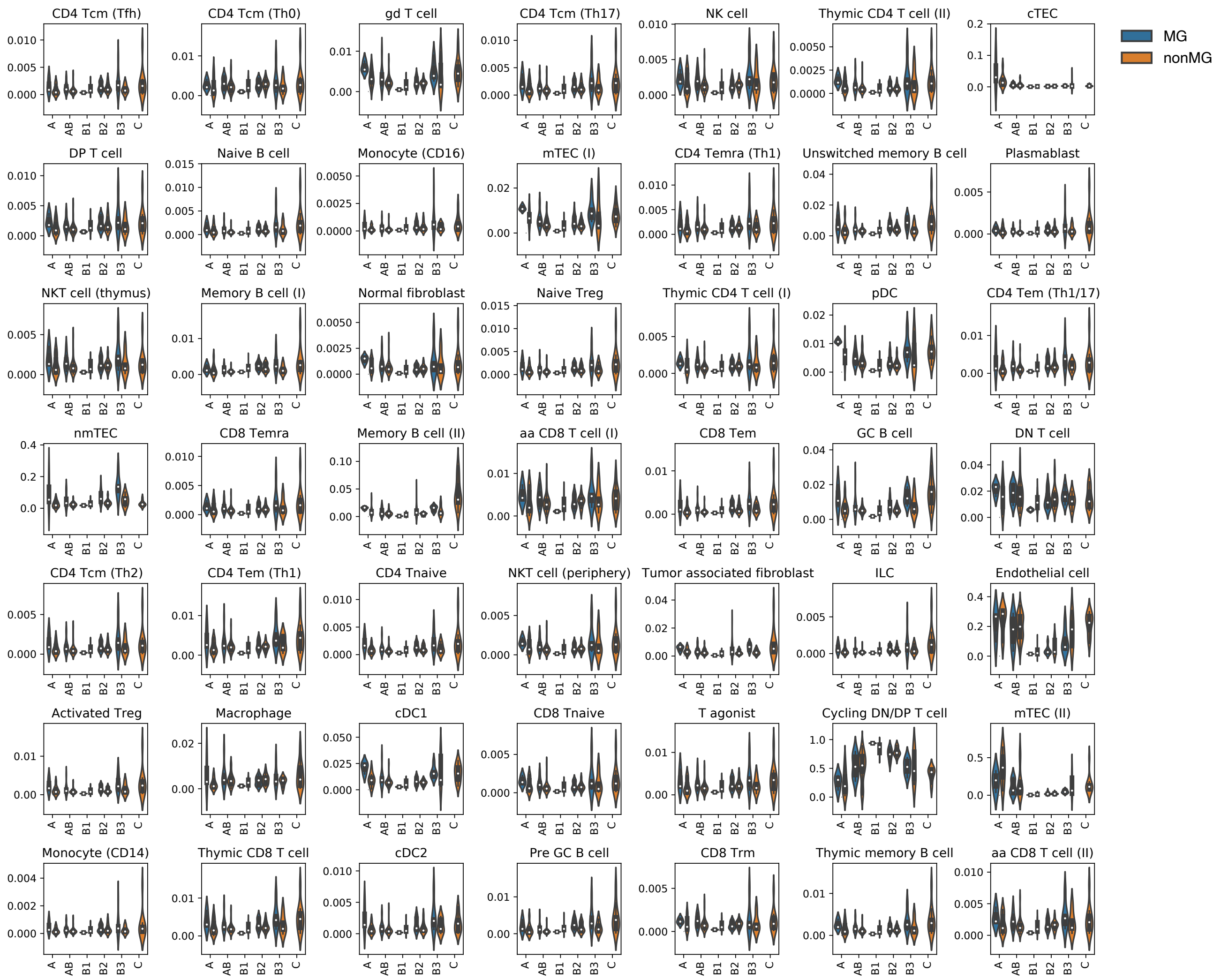

CD4 Tcm (Tfh)
CD4 Tcm (Th0)
gd T cell
CD4 Tcm (Th17)
NK cell
Thymic CD4 T cell (II)
cTEC
DP T cell
Naive B cell
Monocyte (CD16)
mTEC (I)
CD4 Temra (Th1)
Unswitched memory B cell
Plasmablast
NKT cell (thymus)
Memory B cell (I)
Normal fibroblast
Naive Treg
Thymic CD4 T cell (I)
pDC
CD4 Tem (Th1/17)
nmTEC
CD8 Temra
Memory B cell (II)
aa CD8 T cell (I)
CD8 Tem
GC B cell
DN T cell
CD4 Tcm (Th2)
CD4 Tem (Th1)
CD4 Tnaive
NKT cell (periphery)
Tumor associated fibroblast
ILC
Endothelial cell
Activated Treg
Macrophage
cDC1
CD8 Tnaive
T agonist
Cycling DN/DP T cell
mTEC (II)
Monocyte (CD14)
Thymic CD8 T cell
cDC2
Pre GC B cell
CD8 Trm
Thymic memory B cell
aa CD8 T cell (II)
A
AB
B1
B2
B3
C
MG
nonMG

b

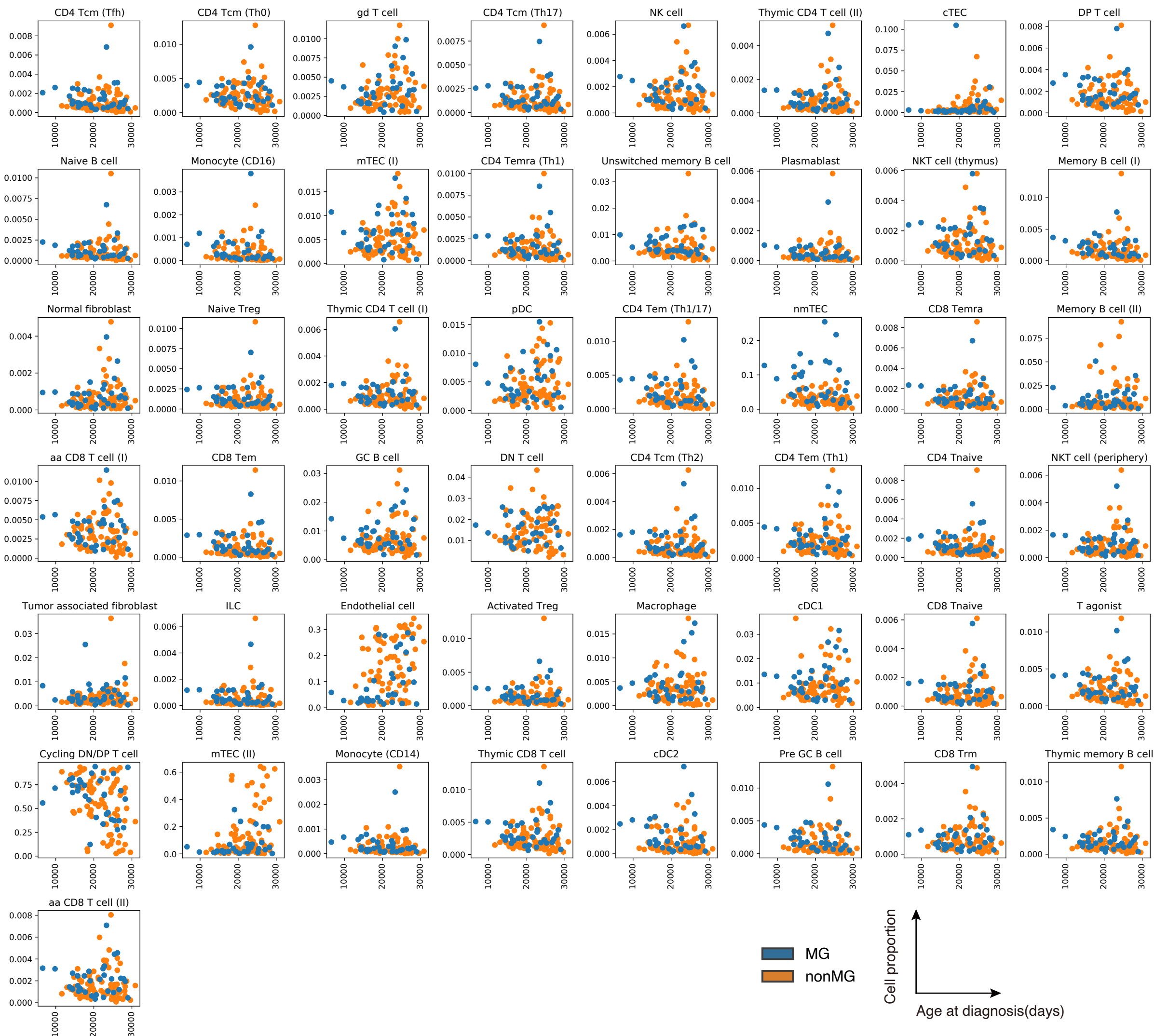

CD4 Tcm (Tfh)
CD4 Tcm (Th0)
gd T cell
CD4 Tcm (Th17)
NK cell
Thymic CD4 T cell (II)
cTEC
DP T cell
Naive B cell
Monocyte (CD16)
mTEC (I)
CD4 Temra (Th1)
Unswitched memory B cell
Plasmablast
NKT cell (thymus)
Memory B cell (I)
Normal fibroblast
Naive Treg
Thymic CD4 T cell (I)
pDC
CD4 Tem (Th1/17)
nmTEC
CD8 Temra
Memory B cell (II)
aa CD8 T cell (I)
CD8 Tem
GC B cell
DN T cell
CD4 Tcm (Th2)
CD4 Tem (Th1)
CD4 Tnaive
NKT cell (periphery)
Tumor associated fibroblast
ILC
Endothelial cell
Activated Treg
Macrophage
cDC1
CD8 Tnaive
T agonist
Cycling DN/DP T cell
mTEC (II)
Monocyte (CD14)
Thymic CD8 T cell
cDC2
Pre GC B cell
CD8 Trm
Thymic memory B cell
aa CD8 T cell (II)
10000
20000
30000
MG
nonMG
Cell proportion
Age at diagnosis(days)

Supplementary Figure 14 Inferred cell proportion in TCGA bulk RNA-seq in thymoma (n=116).

a, Violin plots of deconvoluted cell proportion partitioned by WHO classification and MG status. Box plots show IQRs and whiskers show the maximum or minimum value in the dataset excluding outliers (Q3+1.5×IQR or Q1-1.5×IQR). b, Scatter plot showing the relationship between deconvoluted cell proportion (y-axis) and age at diagnosis (x-axis). Dot color represents MG status. Cell deconvolution analysis was performed for TCGA thymoma bulk RNA-seq samples with single-cell annotations as the reference (a,b). Scaden[47] was used for the deconvolution.

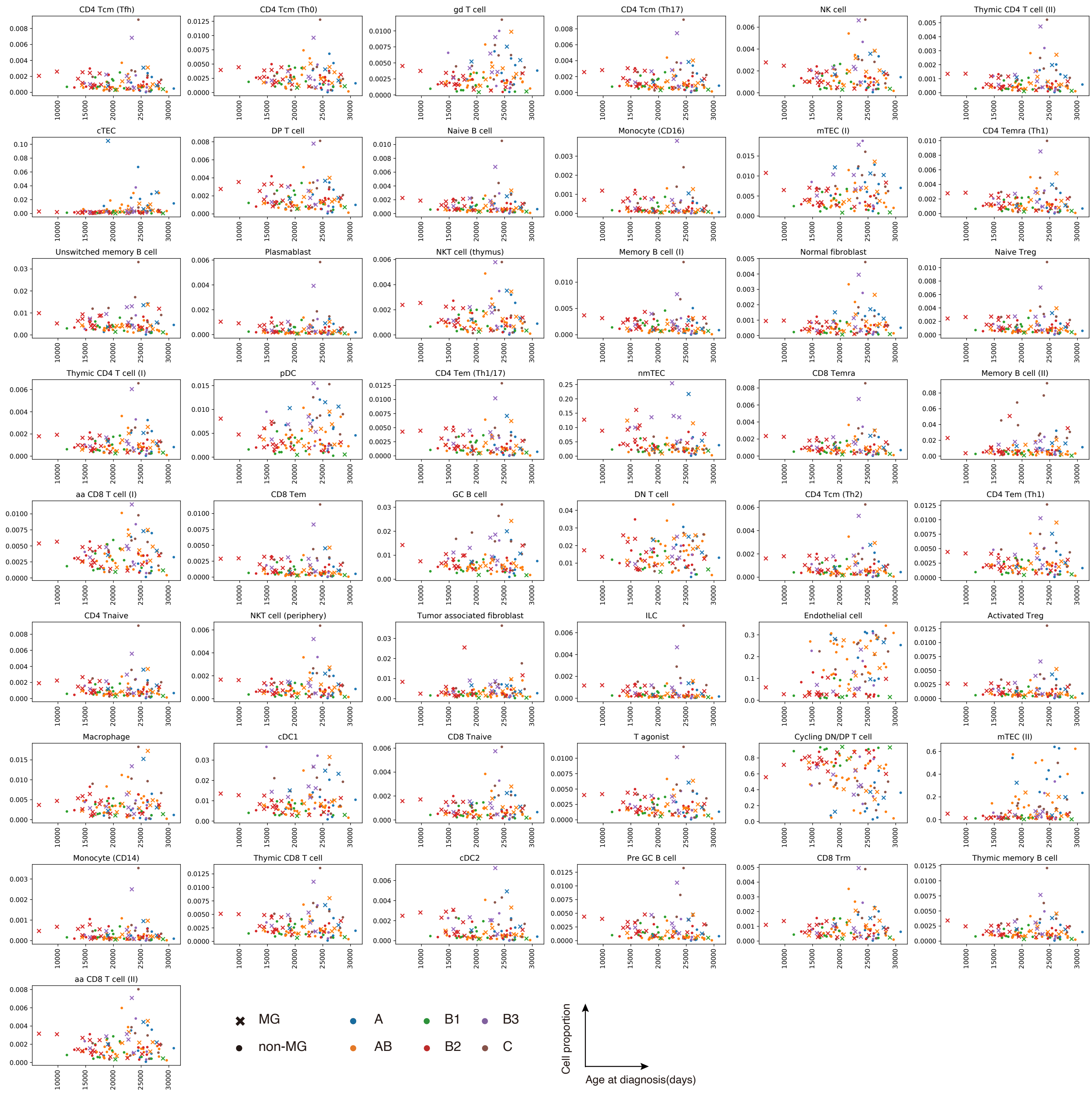

Supplementary Figure 15 The scatter plot showing deconvoluted cell proportion and age at diagnosis. The marker shape represents the disease status, and the color does WHO classification.

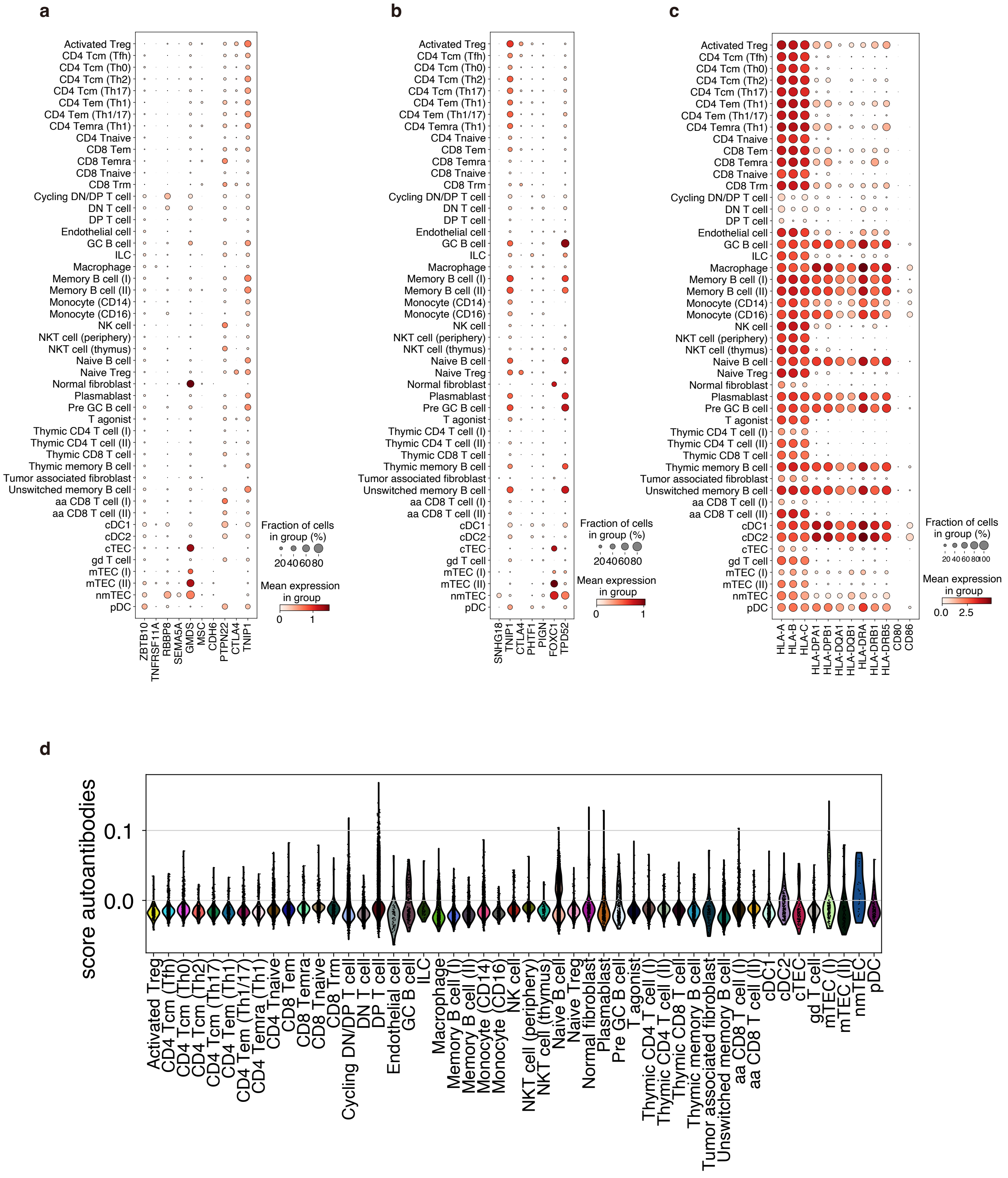

Supplementary Figure 16 Cell-type wide expression of GWAS reported genes, HLA, and targets of autoantibodies thymoma-associated neuromuscular disorders.

a-c, Dot plot of gene expression of GWAS reported genes (a), GWAS genes mapped by eQTL (b), and HLA and costimulatory molecules (c) across cell types. d, Violin plots of the signature score of targets of autoantibodies causing neuromuscular disorders associated with thymoma listed in Supplementary Data sheet 6. Box plots show IQRs and whiskers show the maximum or minimum value in the dataset excluding outliers (Q3+1.5×IQR or Q1-1.5×IQR).
