# Supplementary material for: Myasthenia gravis-specific aberrant neuromuscular gene expression by medullary thymic epithelial cells in thymoma
Source: Nat Commun. 2022 Jul 22;13:4230. doi: 10.1038/s41467-022-31951-8 (PMC9305039; doi:10.1038/s41467-022-31951-8)
Supplement: Supplementary file 3 — Description of Additional Supplementary Files [file 41467_2022_31951_MOESM3_ESM.docx]

**Description of Additional Supplementary Files**

**Supplementary Data 1:**

DEGs MG vs non-MG (TCGA bulk)

Differentially expressed genes in the comparison between MG and nonMG thymoma in TCGA dataset. Statistical tests and multiple test correction were performed using DESeq2. Adjusted p-value < 0.1 were included in the list.

**Supplementary Data 2:**

WGCNA gene modules

Genes included in WGCNA gene modules.

**Supplementary Data 3:**

Gene sets for Yellow (MGup)

REACTOME gene sets enriched in the WGCNA Yellow module, which possessed a significant correlation with MG.

**Supplementary Data 4:**

Gene sets for WGCNA modules

REACTOME gene sets enriched in the WGCNA modules.

**Supplementary Data 5:**

AutoAbs references

Publications used for the creation of the list of targets of autoantibodies.

**Supplementary Data 6:**

Targets of autoantibodies

Curated targets of autoantibodies (Gene Symbol).

**Supplementary Data 7:**

Patient info (scRNAseq)

Patient information for scRNAseq analysis.

**Supplementary Data 8:**

Num cells (scRNAseq)

The number of cells for each cluster in the periphery and the thymoma.

**Supplementary Data 9:**

marker genes (manual)

Manually arranged marker genes.

**Supplementary Data 10:**

scRNAseq Marker genes (200)

The top 200 marker genes for each cluster calculated by the scanpy.tl.rank_genes_groups function.

**Supplementary Data 11:**

REACTOME(nmTEC)

Enriched REACTOME gene sets in nmTEC cells.

**Supplementary Data 12:**

REACTOME(mTECI)

Enriched REACTOME gene sets in mTEC(I) cells.

**Supplementary Data 13:**

REACTOME(mTECII)

Enriched REACTOME gene sets in mTEC(II) cells.

**Supplementary Data 14:**

REACTOME(cTEC)

Enriched REACTOME gene sets in cTEC cells.

**Supplementary Data 15:**

Tissue restricted antigens

Tissue restricted antigens calculated using GTEx datasets (Methods).

**Supplementary Data 16:**

GWAS summary

Curated GWAS publications used for cell-type enrichment analysis.

**Supplementary Data 17:**

cell-type enrichment

The detailed results of cell-type enrichment analysis (Fig. 6d).

**Supplementary Data 18:**

Antibody list

Antibody list used for the histological investigations in this study.
