# Supplementary material for: Myasthenia gravis-specific aberrant neuromuscular gene expression by medullary thymic epithelial cells in thymoma
Source: Nat Commun. 2022 Jul 22;13:4230. doi: 10.1038/s41467-022-31951-8 (PMC9305039; doi:10.1038/s41467-022-31951-8)
Supplement: Supplementary file 22 — Reporting Summary [file 41467_2022_31951_MOESM22_ESM.pdf]

nature portfolio

Corresponding author(s): Naganari Ohkura and Tatsusada Okuno

Last updated by author(s): May 23rd 2022

Reporting Summary

Nature Portfolio wishes to improve the reproducibility of the work that we publish. This form provides structure for consistency and transparency in reporting. For further information on Nature Portfolio policies, see our Editorial Policies and the Editorial Policy Checklist.

Statistics

For all statistical analyses, confirm that the following items are present in the figure legend, table legend, main text, or Methods section.

n/a	Confirmed	
☐	☒	The exact sample size (*n*) for each experimental group/condition, given as a discrete number and unit of measurement
☐	☒	A statement on whether measurements were taken from distinct samples or whether the same sample was measured repeatedly
☐	☒	The statistical test(s) used AND whether they are one- or two-sided<br>*Only common tests should be described solely by name; describe more complex techniques in the Methods section.*
☒	☐	A description of all covariates tested
☐	☒	A description of any assumptions or corrections, such as tests of normality and adjustment for multiple comparisons
☐	☒	A full description of the statistical parameters including central tendency (e.g. means) or other basic estimates (e.g. regression coefficient) AND variation (e.g. standard deviation) or associated estimates of uncertainty (e.g. confidence intervals)
☐	☒	For null hypothesis testing, the test statistic (e.g. *F*, *t*, *r*) with confidence intervals, effect sizes, degrees of freedom and *P* value noted<br>*Give P values as exact values whenever suitable.*
☐	☒	For Bayesian analysis, information on the choice of priors and Markov chain Monte Carlo settings
☒	☐	For hierarchical and complex designs, identification of the appropriate level for tests and full reporting of outcomes
☐	☒	Estimates of effect sizes (e.g. Cohen's *d*, Pearson's *r*), indicating how they were calculated

Our web collection on statistics for biologists contains articles on many of the points above.

Software and code

Policy information about availability of computer code

Data collection	Cell collection were performed using FACS Aria II. Single-cell RNAseq was performed on NovaSeq6000 (Illumina). Florescent image capture was performed on Zeiss LSM 710 or LSM 880 confocal microscope.
Data analysis	Following tools were used in the analysis; scikit-image (0.18.1), gdc-client(v1.5.0), TCGAbiolinks(2.16.4), DESeq2 (1.30.1), EnhancedVolcano (1.6.0), pheatmap(1.0.12), ggplot2(3.3.5), WGCNA(1.71), clusterProfiler(v3.16.1), ReactiomePA(v1.32.0), STAR (v2.7.2a), arcasHLA (v0.2.0), VIRTUS (v1.2.1), maftools, Cell Ranger (v5.0.0), Scanpy (1.7.2), Python (3.8.0), BBKNN, velocyto, scVelo (0.2.3), seaborn (0.11.1), matplotlib (3.4.1), gesapy, pymc3 (3.11.2), CellPhoneDB (2.1.7), CellPhoneDB (2.1.7), statsmodels (0.12.0), pingouin (0.3.8), Cytoscape (3.8.0).<br>The codes used for this paper were deposited in https://github.com/yyoshiaki/MG_thymoma_Manuscript_2021.

For manuscripts utilizing custom algorithms or software that are central to the research but not yet described in published literature, software must be made available to editors and reviewers. We strongly encourage code deposition in a community repository (e.g. GitHub). See the Nature Portfolio guidelines for submitting code & software for further information.

Data

Policy information about availability of data

All manuscripts must include a data availability statement. This statement should provide the following information, where applicable:

- Accession codes, unique identifiers, or web links for publicly available datasets
- A description of any restrictions on data availability
- For clinical datasets or third party data, please ensure that the statement adheres to our policy

The raw sequence data for single-cell RNA-seq analysis was deposited in JGA (JGAS000482). The processed single-cell RNA-seq data was deposited in Single Cell Portal (https://singlecell.broadinstitute.org/single_cell/study/SCP1532). TCGA data is available on dbGaP accession phs000178. H5ad files of scRNAseq data

previously reported were downloaded respectively (PBMC: https://atlas.fredhutch.org/data/nygc/multimodal/pbmc_multimodal.h5seurat; normal thymus: 10.5281/zenodo.3711134).

Field-specific reporting

Please select the one below that is the best fit for your research. If you are not sure, read the appropriate sections before making your selection.

- [x] Life sciences
- [ ] Behavioural & social sciences
- [ ] Ecological, evolutionary & environmental sciences

For a reference copy of the document with all sections, see nature.com/documents/nr-reporting-summary-flat.pdf

Life sciences study design

All studies must disclose on these points even when the disclosure is negative.

Sample size	Since this is a preclinical study, no statistical measures were conducted to predetermine the sample size. All sample size, statistical tests and p-values are indicated in the figure legends and described in methods.
Data exclusions	No data were excluded from analysis.
Replication	Histological experiments were performed with at least three independent replicates as indicated in the figure legends and methods. All data was taken from single experiment.
Randomization	Sample selection for histological verification of the KRT expressions and the co-localization of CD31 and GABRA5 were performed randomly.
Blinding	Data acquisition and analysis in these studies were performed in a blinded way.

Reporting for specific materials, systems and methods

We require information from authors about some types of materials, experimental systems and methods used in many studies. Here, indicate whether each material, system or method listed is relevant to your study. If you are not sure if a list item applies to your research, read the appropriate section before selecting a response.

Materials & experimental systems

n/a	Involved in the study	
[ ]	[x]	Antibodies
[x]	[ ]	Eukaryotic cell lines
[x]	[ ]	Palaeontology and archaeology
[x]	[ ]	Animals and other organisms
[ ]	[x]	Human research participants
[x]	[ ]	Clinical data
[x]	[ ]	Dual use research of concern

Methods

n/a	Involved in the study	
[x]	[ ]	ChIP-seq
[ ]	[x]	Flow cytometry
[x]	[ ]	MRI-based neuroimaging

Antibodies

Antibodies used

clone : For histological experiments, we used following antibodies;
Antigen Gene Clone Host Class Concentration Supplier
CD11C ITGAX 5D11 Mouse IgG1 1/100 Novocastra
CD31 PECAM1 JC70A Mouse IG1 1/200 Agilent Technologies
CD79A CD79A JCB117 Mouse IgG1 1/200 Agilent Technologies
NF 160kda NEFM NN18 Mouse IgG1 1/500-1/1000 Sigma-Aldrich
KRT6 KRT6C sc-514520 Mouse IgG1 1/100 Santa Cruz
KRT17 KRT17 sc-393002 Mouse IgG2 1/100 Santa Cruz
TF TF sc-365871 Mouse IgG1 1/50 Santa Cruz
SOX15(c-7) SOX15 sc-166964 Mouse IgM 1/50 Santa Cruz
MAP2 MAP2 sc-74421 Mouse IgG1 1/100 Santa Cruz
GLI2 GLI2 sc-271786 Mouse IgG1 1/100 Santa Cruz
NEFL NEFL sc-20012 Mouse IgG1 1/25 Santa Cruz
GABRA5 GABRA5 sc-393921 Mouse IgG1 1/25 Santa Cruz

For FACS sorting, we used the following antibodies;
FITC-labeled anti-EpCAM mAb (dilution: 1/10, clone : HEA-125, Miltenyi Biotec)
PE-labeled anti-CD45 mAb (dilution: 1/100, clone : HI30, Biolegend)
FITC-labeled anti-CD3 mAb (dilution: 1/100, clone : UCHT1, BD Bioscience)

	APC-labeled anti-CD4 mAb (dilution: 1/100, clone : RPA-T4, Thermo Fisher Scientific)<br>PE-labeled anti-CD19 mAb (dilution: 1/100, clone : HIB19, BioLegend)
Validation	All antibodies used in this study were previously confirmed by the manufacturers and previous research studies that can be found on the manufacturers' websites.

Human research participants

Policy information about studies involving human research participants

Population characteristics	All participants were Japanese. FFPE specimens of thymoma (WHO classification AB-B3, with/without MG) from patients (age 26-81) who experienced thymectomy were used for histological investigations (Source Data). PBMC and resected thymoma samples from thymoma patients with MG (age 35-55) were used for single-cell RNA-seq analysis. Detailed patient profiles (age, gender, treatment, and anti-AchR Abs titer) were described in the supplemental information.
Recruitment	Patients who experienced thymectomy without any chemotherapy were included in histological investigations. Patients who experienced thymectomy and are with MG were included in the single-cell RNA-seq analysis.
Ethics oversight	Human samples were collected under approved Osaka University's review board protocols: ID 10038-9 and ID 850-2. Written informed consent was obtained from all donors.

Note that full information on the approval of the study protocol must also be provided in the manuscript.

Flow Cytometry

Plots

Confirm that:

- [x] The axis labels state the marker and fluorochrome used (e.g. CD4-FITC).
- [x] The axis scales are clearly visible. Include numbers along axes only for bottom left plot of group (a 'group' is an analysis of identical markers).
- [x] All plots are contour plots with outliers or pseudocolor plots.
- [x] A numerical value for number of cells or percentage (with statistics) is provided.

Methodology

Sample preparation	To ensure the quality of the library, the library preparation of all thymoma and peripheral blood samples was completed by the next day after the collection. Immune cells and thymic epithelial cells were isolated from thymic tissue dissected surgically, as previously described. Briefly, thymic tissue was mechanically disrupted, and the fraction containing lymphocytes was collected. Extracted cells were stained with 7-AAD (BD Biosciences), and live cells were collected as a lymphocyte fraction. The remaining thymic tissue was subjected to enzymatic treatment (Collagenase A (Worthington), DNAse I (Roche, Basel Switzerland), Trypsin/EDTA (nacalai tesque)) and the resulting cells were then subjected to a percoll density gradient centrifugation for the enrichment of thymic epithelial cells. Cells derived from low-density fraction were stained using FITC-labeled anti-EpCAM mAb (dilution: 1/10, HEA-125, Miltenyi Biotec), PE-labeled anti-CD45 mAb (dilution: 1/100, HI30, Biolegend). Dead cells were excluded by 7-AAD staining, and CD45 (low) EpCAM (high) was defined as thymic epithelial cells. Immune cells and thymic epithelial cells were isolated using BD Biosciences FACS Aria II. The gating strategy is described in Supplementary Data Fig. 3. For CD4+ T cells and B cells, we first collected PBMCs using Ficoll-Paque (Cytiva). Isolated PBMCs were washed, blocked Fc receptors using Fc Receptor Binding Inhibitor Polyclonal Antibody, Functional Grade, eBioscience™ (Thermo Fisher Scientific), and stained using FITC-labeled anti-CD3 mAb (dilution: 1/100, UCHT1, BD Bioscience), APC-labeled anti-CD4 mAb (dilution: 1/100, RPA-T4, Thermo Fisher Scientific), PE-labeled anti-CD19 mAb (HIB19, BioLegend), Live/Dead (Thermo Fisher Scientific). Then, live-CD3+CD4+CD19- cells and live-CD3-CD4-CD19+ cells were isolated using BD Biosciences FACS Aria II.
Instrument	BD Biosciences FACS Aria II
Software	Cells were analyzed using FACSAria II. Data analysis was done using Flow Jo.
Cell population abundance	Sorted cells were used for single-cell RNA-seq experiments, and re-annotated based on the gene expression profiles manually as shown in the paper.
Gating strategy	Immune cells : live-cells<br>Thymic epithelial cells : live-CD45loEPICAMhi cells<br>CD4T cells : live-CD3+CD4+CD19- cells<br>B cells : live-CD3-CD4-CD19+ cells were isolated

- [x] Tick this box to confirm that a figure exemplifying the gating strategy is provided in the Supplementary Information.
